# A pair of new enantiomers of xanthones from the stems and leaves of *Cratoxylum cochinchinense*

**DOI:** 10.1186/s13020-019-0235-z

**Published:** 2019-03-29

**Authors:** Cuicui Jia, Chi Gong, Hong Chen, Jing Pu, Dahong Li, Zhanlin Li, Huiming Hua

**Affiliations:** 1 Key Laboratory of Structure-Based Drug Design & Discovery, Ministry of Education, Shenyang Pharmaceutical University, Shenyang, 110016 Liaoning People’s Republic of China; 2 Department of Psychiatry, Qiqihar Medical University, Qiqihar, 161006 Heilongjiang People’s Republic of China; 3 School of Traditional Chinese Materia Medica, Shenyang Pharmaceutical University, Shenyang, People’s Republic of China; 4 School of Life Science and Biopharmaceutics, Shenyang Pharmaceutical University, Shenyang, People’s Republic of China

**Keywords:** *Cratoxylum cochinchinense*, Xanthone, Antitumor, Enantiomer

## Abstract

**Background:**

The simple and caged xanthones from Clusiaceae showed significant antineoplastic activity. This study aims to identify structural diverse xanthones and search for novel antitumor natural products from this family plants.

**Methods:**

The structures of new compounds **1a** and **1b** were elucidated mainly through comprehensive NMR and MS spectroscopic data, and their absolute configurations were determined by the comparison of the experimental and calculated electronic circular dichroism.

**Results:**

A pair of new xanthone enantiomers, (+)- and (−)-cracochinxanthone A (**1a** and **1b**), along with thirty known analogues (**2**–**31**), were isolated from extracts of the stems and leaves of *C. cochinchinense.* Preliminary biological assay of some isolates against HL-60, PC-3, and MDA-MB-231 cancer cell lines.

**Conclusion:**

Some isolated xanthones exhibited high sensitivity against three human malignant cell lines and the structure–activity relationship study showed that the prenyl and geranyl units may play an important role in antitumor activity.

**Electronic supplementary material:**

The online version of this article (10.1186/s13020-019-0235-z) contains supplementary material, which is available to authorized users.

## Background

**Fig. 1:**
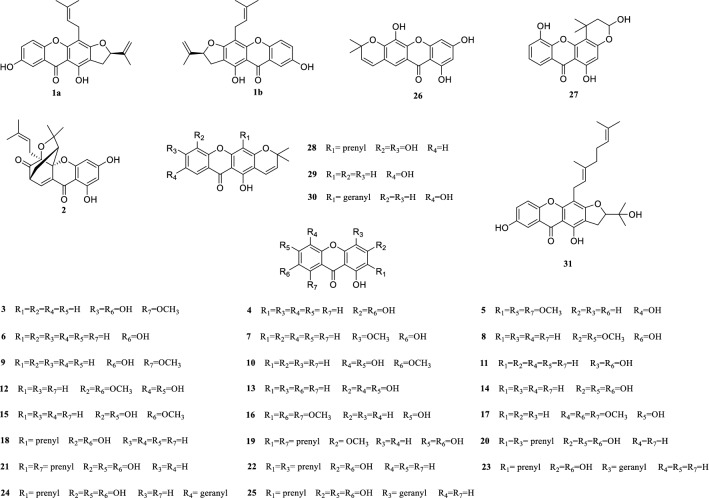
Chemical structures of xanthones **1**–**31**

*Cratoxylum cochinchinense* Blume (Clusiaceae) is a deciduous shrub tree growing abundantly in southeast Asian countries [1]. The leaves, stems, barks, roots and latex of *C. cochinchinense* have been used as traditional Chinese medicine for the treatment of various diseases such as jaundice, edema, cough, itch, fever, diarrhea, hoarseness, diuretic, flu, colic, ulcer and dental problems and so on [2–4]. In addition, the young leaves have been used as an herbal substitute for tea and the immature fruit as a spice for cooking [5]. The simple and caged xanthones with significant antineoplastic activity have been reported from previous phytochemical investigations [6–12]. Aiming to identify structural diverse xanthones and search for novel antitumor natural products from the Clusiaceae [13–18], we continued our studies on the petroleum ether-soluble and dichloromethane-soluble portions of the stems and leaves of *C. cochinchinense* which exhibited moderate cytotoxicity against human myeloid leukemia (HL-60), human prostate cancer (PC-3) and human breast carcinoma (MDA-MB-231) cell lines with IC_50_ values of 7.59, 21.49, 19.63 and 7.86, 32.48, 30.40 μg/ml, respectively. A pair of new enantiomers of xanthones, (+)- and (−)-cracochinxanthone A (**1a** and **1b**), as well as thirty known analogues (**2–31**) were obtained (Fig. 1). In the present paper, the isolation and structure elucidation of new enantiomers of **1a** and **1b**, as well as the biological evaluation of some selected xanthones are presented.

## Materials and methods

### Information of experimental design and resources

The Minimum Standards of Reporting Checklist contains details of the experimental design, and statistics, and resources used in this study (Additional file 1).

### General experimental procedures

^1^H NMR, ^13^C NMR, HSQC, and HMBC were recorded on the Bruker-ARX-400 and Bruker-AV-600 NMR with tetramethylsilane (TMS) as internal standard. HRESIMS spectra were measured on a Bruker micrOTOF-Q mass spectrometer. Optical rotations were measured by the JASCO P-2000 polarimeter. UV spectra were recorded on a Shimadzu UV-2201 spectrometer. ECD spectra were measured on the BioLogic MOS 450 AF/CD at room temperature. Multimode Reader were used by a Varioskan Flash. The semi-preparative HPLC was a Shimadzu SPD-20A series equipped with an YMC C_18_ column (250 × 20 mm, 5 μm, 2 mL/min). Chiral HPLC was a CHIRALPAK IB (250 × 4.6 mm) from Daicel Chiral Technologies Co., Ltd., China. Column chromatography (CC) was conducted on silica gel (100–200 and 200–300 mesh) and preparative and analytical TLC was performed on precoated GF254 plates (Qingdao Haiyang Chemical Co., Ltd., China), octadecyl silane (ODS) (50 µm, YMC Co., Ltd., Kyoto, Japan) and Sephadex LH-20 (GE Healthcare, Uppsala, Sweden). All the organic solvents were purchased from Yuwang and Laibo Chemicals Industries, Ltd., China.

### Plant material

Stems and leaves of *Cratoxylum cochinchinense* were collected in December 2016, at Mengla County, Xishuangbanna Autonomous Prefecture, People’s Republic of China, and were identified by Zhi Na (Kunming Institute of Botany, Chinese Academy of Sciences). The voucher specimen (HNMJY-2016) was deposited in the Department of Natural Products Chemistry, Shenyang Pharmaceutical University, Shenyang, China.

### Extraction and isolation

The smashed leaves and stems of *C. cochinchinense* (10 kg) were macerated with 80% aqueous acetone at room temperature (3 × 80 L, 3 days each time). The combined extracts was suspended in water, and successively partitioned to produce petroleum ether (PE), dichloromethane (CH_2_Cl_2_), ethyl acetate (EtOAc), *n*-butyl alcohol (*n*-BuOH) and water (H_2_O) fractions. The CH_2_Cl_2_ extract (140 g) was fractionated on a silica gel CC and eluted with a PE/EtOAc gradient (100:0, 100:1, 100:3, 100:5, 100:7, 100:10, 100:20, 100:30, 100:50, 0:100) to give ten fractions (Fr. A–J). Fraction D was purified by ODS CC with a stepwise gradient elution using MeOH/H_2_O to afford **23** (154.8 mg), **29** (8.2 mg), **22** (5.8 mg), **26** (8.7 mg) and yield two subfractions D3 and D4. Fr. D3 was subsequently refined over Sephadex LH-20 (MeOH), followed by semi-preparative HPLC using 77% MeOH in H_2_O as a mobile phase to get **19** (15.8 mg, t_R_ = 63.5 min) and **1** (3.2 mg, t_R_ = 93.3 min). Then, **1** was separated by chiral HPLC eluting with *n*-hexane: isopropanol (90:10) to yield **1a** (0.92 mg, t_R_ = 15.4 min) and **1b** (1.1 mg, t_R_ = 18.4 min). Fr. D4 was also chromatographed on Sephadex LH-20 (MeOH) and semi-preparative HPLC (64% MeOH in H_2_O) to produce **6** (4.5 mg, t_R_ = 38.4 min) and **9** (6.5 mg, t_R_ = 40.3 min). Fr. F was fractionated by ODS CC (MeOH/H_2_O) to give **27** (7.5 mg), **18** (5.3 mg), **20** (7.9 mg) and three major subfractions F2, F5 and F8. Fr. F2 was successively partitioned by a Sephadex LH-20 column (MeOH) to provide the key subfraction F2.2. Fr. F2.2 was further processed via semi-preparative HPLC using 56% aqueous MeOH as the mobile phase to afford **3** (10.2 mg, t_R_ = 28.0 min) and **4** (7.8 mg, t_R_ = 32.2 min). Fr. F5 was recrystallized with methanol to yield **19** (50.1 mg). Fr. F8 was loaded onto semi-preparative HPLC using 82% aqueous MeOH to gain **24** (6.9 mg, t_R_ = 87.5 min), **25** (10.2 mg, t_R_ = 92.5 min) and **21** (15.2 mg, t_R_ = 121.5 min). Fr. H was subjected to ODS CC, which afford **15** (6.9 mg) through further recrystallization and subfractions H3 and H5. Fr. H3 and Fr. H5 were applied to Sephadex LH-20 column and eluted with MeOH to obtain **7** (5.4 mg) and **8** (7.5 mg), respectively. Fr. I was subjected to ODS CC to furnish **13** (5.2 mg), **14** (2.8 mg), and subfraction I4. Fr. I4 followed by Sephadex LH-20 CC to afford **28** (11.6 mg). Fr. J was rechromatographed over silica gel CC, affording **12** (4.6 mg), **11** (3.2 mg) and **10** (13.4 mg).

The PE extract (69 g) was chromatographed on a silica gel CC and eluted stepwise with a PE/EtOAc gradient system (100:1, 100:3, 100:7, 100:15, 100:50, 100:100, 0:100) to afford the major fractions A′-G′. Fr. C′ was subjected to separation over ODS CC to yield **30** (8.8 mg) and subfraction C′8. Fr. C′8 was further purified over a silica gel CC and followed by semi-preparative HPLC with 90% aqueous MeOH as mobile phase under isocratic condition to furnish **31** (10.7 mg, t_R_ = 25.5 min). Fr. D′ was separated via ODS CC to provide **2** (15.5 mg), which was crystallized from the 65% MeOH/H_2_O solution, and to give subfraction D′5. Fr. D′5 was chromatographed over Sephadex LH-20 eluting with MeOH to give **17** (6.7 mg). Fr. E′ was initially subjected to ODS CC to yield subfraction E′3 and E′8. Fr. E′3 was further purified by semi-preparative HPLC eluted with 60% MeOH/H_2_O to give **16** (9.3 mg, t_R_ = 35.8 min). Fr. E′8 was again subjected to ODS CC to obtain **5** (3.1 mg).

Cracochinxanthone A **(1)**: yellow needle crystal; UV (MeOH) *λ*_max_ (log ε) 319 (3.86), 268 (4.23), 235 (4.21) nm; ^1^H, ^13^C NMR and HMBC data see Table 1; HRESIMS *m/z* 379.1541 [M + H]^+^ (calcd for C_23_H_23_O_5_, 379.1540).

**Table 1:** ^1^H (600 MHz), ^13^C NMR (150 MHz) and HMBC data for compound **1** in DMSO-*d*_6_

Position	^1^H-NMR (*mult, J in Hz*)	^13^C–NMR	HMBC (^1^H → ^13^C)
1		158.0	
2		108.0	
3		163.5	
4		106.3	
5	7.46 (1H, d, *J *= 9.0 Hz)	118.6	C-7, 8a, 10a
6	7.28 (1H, dd, *J *= 9.0, 3.0 Hz)	124.4	C-8, 10a
7		153.8	
8	7.40 (1H, d, *J *= 3.0 Hz)	107.9	C-6, 9, 10a
9		179.9	
4a		152.9	
8a		120.1	
9a		101.5	
10a		149.0	
1′	2.95 (1H, dd, *J *= 14.7, 3.1 Hz) 2.82 (1H, dd, *J *= 14.7, 8.0 Hz)	28.9	C-1, 2, 3, 2′, 3′
2′	4.24 (1H, dd, *J *= 8.0, 2.3 Hz)	74.8	C-2, 1′, 3′
3′		147.1	
4′	4.89 and 4.75 (each 1H, s)	110.0	C-2′, 3′, 5′
5′	1.76 (3H, s)	18.1	C-2′, 3′, 4′
1″	3.43 (2H, d, *J *= 7.2 Hz)	21.6	C-3, 4, 4a, 2″, 3″
2″	5.19 (1H, t, *J *= 7.2 Hz)	122.5	C-1″, 4″, 5″
3″		130.6	
4″	1.82 (3H, s)	17.8	C-2″, 3″, 5″
5″	1.62 (3H, s)	25.6	C-2″, 3″
1-OH	13.35 (1H, s)		C-1, 2, 9a
7-OH	9.95 (1H, s)		C-6, 7, 8

(+) Cracochinxanthone A (**1a**). Yellow needles; $\left[ \alpha \right]_{\text{D}}^{25}$ + 10.0 (*c* 0.06 MeOH); ECD (MeOH 0.58) *λ*_max_ (Δ*ε*) 241 (+ 3.65), 270 (− 4.22), 317 (− 1.91) nm.

(−) Cracochinxanthone A (**1b**). Yellow needles; $\left[ \alpha \right]_{\text{D}}^{25}$ − 11.3 (*c* 0.07 MeOH); ECD (MeOH 0.70) *λ*_max_ (Δ*ε*) 242 (− 4.06), 273 (+ 3.72), 316 (+ 1.45) nm.

### Anticancer assay in vitro

The antiproliferative activities of some selected compounds against the HL-60, PC-3, and MDA-MB-231 cancer cell lines were evaluated. 5-Fluorouracil (5-FU) was used as a positive control. Detailed methodology for the cell growth inhibition test has been described in a previous report [19]. The IC_50_ values were calculated by SPSS 16.0 software and results were repeated three times that were expressed as mean ± SD.

## Results

**Fig. 2:**
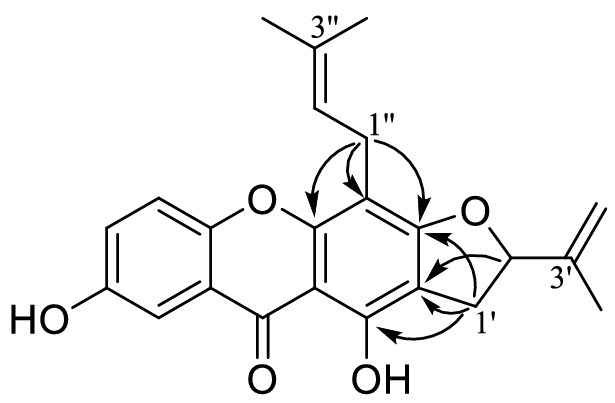
Key HMBC correlations of **1**

Cracochinxanthone A (**1**) was obtained as a yellow needle, and its molecular formula was determined as C_23_H_22_O_5_ with 13° of unsaturation from the HRESIMS data of [M + H]^+^ ion at *m/z* 379.1541 (calcd for C_23_H_23_O_5_, 379.1540). The UV bands observed at *λ*_max_ 319, 268 and 235 nm suggested a xanthone skeleton [13]. The ^1^H NMR data showed signals for a hydrogen bond hydroxy proton at *δ*_H_ 13.35 (1H, s, OH-1), a free phenolic hydroxy proton at *δ*_H_ 9.95 (1H, s, OH-7), a set of ABX coupling system aromatic protons at *δ*_H_ 7.40 (1H, d, *J *= 3.0 Hz, H-8), 7.46 (1H, d, *J *= 9.0 Hz, H-5) and 7.28 (1H, dd, *J *= 9.0, 3.0 Hz, H-6), along with the typical signals of a 3-methylbut-2-enyl (prenyl) moiety at *δ*_H_ 3.43 (2H, d, *J *= 7.2 Hz, H-1″), 5.19 (1H, t, *J *= 7.2 Hz, H-2″), 1.82 (3H, s, CH_3_-4″) and 1.62 (3H, s, CH_3_-5″). The remaining proton signals were assigned to a dihydrofuran ring with an isopropenyl group at *δ*_H_ 2.95 (1H, dd, *J *= 14.7, 3.1 Hz, Ha-1′), 2.82 (1H, dd, *J *= 14.7, 8.0 Hz, Hb-1′), 4.24 (1H, dd, *J *= 8.0, 2.3 Hz, H-2′), 4.89 and 4.75 (each 1H, s, Ha-4′, Hb-4′), 1.76 (3H, s, CH_3_-5′), and the corresponding carbon signals at *δ*_C_ 147.1 (C-3′), 110.0 (C-4′), 74.8 (C-2′), 28.9 (C-1′) and 18.1 (C-5′) were assigned through HSQC correlations [20]. The ^13^C NMR displayed 23 carbon resonances including one conjugated carbonyl carbon, sixteen aromatic/olefinic carbons, three methyl, two methylene and one oxygenated methine (Table 1). The dihydrofuran ring with an isopropenyl group was fused with xanthone skeleton at position C-2 and C-3, based on the HMBC correlations from Ha-1′ (*δ*_H_ 2.95) and Hb-1′ (2.82) to C-1 (*δ*_C_ 158.0), C-2 (108.0) and C-3 (163.5), as well as from H-2′ (*δ*_H_ 4.24) to C-2. The cross peaks between H-1″ (*δ*_H_ 3.43) and C-3, C-4a (*δ*_C_ 152.9) and C-4 (106.3) confirmed the location of the prenyl group at C-4. The correlations of H-5 (*δ*_H_ 7.46) with C-7 (*δ*_C_ 153.8), C-8a (120.1) and C-10a (149.0), H-8 (*δ*_H_ 7.40) with C-6 (124.4), C-9 (*δ*_C_ 179.9) and C-10a, H-6 (*δ*_H_ 7.28) with C-8 (*δ*_C_ 107.9) and C-10a, and OH-7 (*δ* 9.95) with C-6, C-7 and C-8 indicated that the free phenolic hydroxy located at C-7 (Fig. 2). Based on these results, the structure of **1** was assigned to a new compound, namely cracochinxanthone A.

**Fig. 3:**
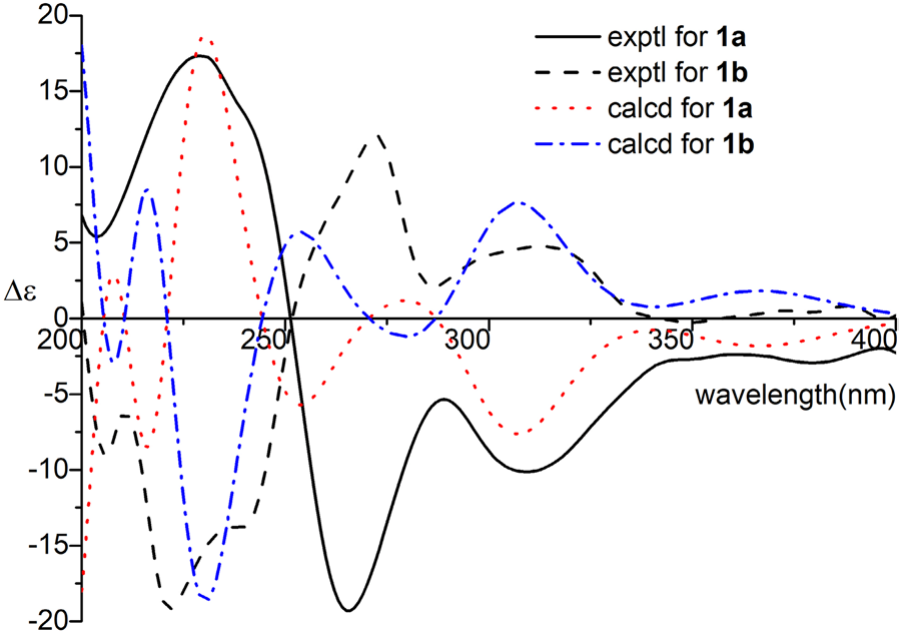
Experimental and calculated ECD spectra of **1a** and **1b**

Cracochinxanthone A might be a racemic mixture due to the smooth ECD curve as well as close to zero optical rotation. Subsequent chiral HPLC separation of **1** gave the corresponding enantiomers **1a** and **1b** possessing the opposite ECD curves. Their experimental ECD spectra matched well with the calculated ones for *R* and *S*, respectively, thus, explicitly assigning the absolute configurations of **1a** and **1b** (Fig. 3). And the optical rotations of **1a** and **1b** were + 10.0 (*c* 0.06 MeOH) and − 11.3 (*c* 0.07 MeOH), respectively. Therefore, the structures of **1a** and **1b** were named as (+) and (−)-cracochinxanthone A.

By comparison with those data from the literatures, the known analogues were identified as cochinchinoxanthone (**2**) [21], 1,4,7-trihydroxy-8-methoxyxanthone (**3**) [22], gentisein (**4**) [23], 1,6-dihydroxy-2,5,8-trimethoxyxanthone (**5**) [23], 1,7-dihydroxyxanthone (**6**) [24], 1,7-dihydroxy-4-methoxyxanthone (**7**) [25], 1,7-dihydroxy-3,6-dimethoxyxanthone (**8**) [26], 1,7-dihydroxy-8-methoxyxanthone (**9**) [27], 1,5,6-trihydroxy-7-methoxyxanthone (**10**) [28], 1,4,7-trihydroxyxanthone (**11**) [29], 1,5,6-trihydroxy-3,7-dimethoxyxanthone (**12**) [30], 1,3,5,6-tetrahydroxyxanthone (**13**) [31], 1,3,6,7-tetrahydroxyxanthone (**14**) [32], 1,3,6-trihydroxy-7-methoxyxanthone (**15**) [29], cratoxanthone C (**16**) [33], 1,2,4-trimethoxy-3,8-dimethoxyxanthone (**17**) [34], 1,3,7-trihydroxy-2-(3-methylbut-2-enyl)-xanthone (**18**) [35], dulcisxanthone B (**19**) [20], cudratricusxanthone E (**20**) [36], γ-mangostin (**21**) [37], 1,3,7-trihydroxy-2,4-diisoprenylxanthone (**22**) [38], cochinchinone A (**23**) [33], cochinchinone B (**24**) [33], pruniflorone Q (**25**) [39], 1,3,5-trihydroxy-6′,6′-dimethyl-2*H*-pyrano(2′,3:6,7)xanthone (**26**) [30], pruniflorone N (**27**) [40], xanthone V_1_ (**28**) [41], osajaxanthone (**29**) [42], cochinchinone I (**30**) [43], 1,7-dihydroxy-4-(3,7-dimethylocta-2,6-dienyl)-5′-(1-hydroxy-1-methylethyl)-4′,5′-dihydrofuro[2′,3′:3,2]-xanthone (**31**) [44].

## Discussions

The antiproliferative activities of some xanthones were evaluated against HL-60, PC-3, and MDA-MB-231 cancer cell lines (Table 2). The isolates **1a**, **1b**, **3**, **4**, **6**, **10**, **12**, **19**–**25**, **27** and **28** displayed antiproliferative effect against HL-60 cells with IC_50_ values ranging from 1.00 to 19.78 μM, especially **23** bearing one prenyl and one geranyl groups with an IC_50_ value of 1.0 μM and **27** possessing a pyran ring with 1-hydroxy-4,4-dimethyl with an IC_50_ value of 1.89 μM. Compounds **19**–**25**, **27** and **28** exhibited potent inhibitory activity against PC-3 cells with IC_50_ values ranging from 11.77 to 27.11 μM and compounds **1b**, **19**–**25** and **28** displayed significant cytotoxicity against MDA-MB-231 cells with IC_50_ values ranging from 7.94 to 18.46 μM, respectively. It is worth to mention that compounds **23**–**25** possessing one prenyl and one geranyl and **19**–**22** with two prenyl groups showed high sensitivity against three human cancer cell lines than others without prenyl unit.

**Table 2:** Cytotoxicities of selected compounds (IC_50_ μM)

Compounds	HL-60	PC-3	MDA-MB-231
**1a**	12.08 ± 0.84	> 50	> 50
**1b**	19.24 ± 1.51	> 50	18.46 ± 1.65
**3**	19.78 ± 2.09	> 50	> 50
**4**	18.00 ± 1.04	> 50	> 50
**6**	15.56 ± 0.51	> 50	> 50
**9**	> 50	> 50	> 50
**10**	10.43 ± 0.31	> 50	> 50
**11**	> 50	> 50	> 50
**12**	10.77 ± 0.13	> 50	> 50
**19**	2.62 ± 0.74	21.87 ± 1.94	7.94 ± 0.94
**20**	4.50 ± 0.17	11.77 ± 0.19	11.97 ± 0.65
**21**	3.07 ± 0.16	27.11 ± 1.49	13.30 ± 1.09
**22**	9.64 ± 0.34	20.60 ± 1.64	14.59 ± 1.26
**23**	1.00 ± 0.21	11.95 ± 1.36	9.40 ± 1.28
**24**	6.18 ± 0.31	14.99 ± 1.28	15.96 ± 0.46
**25**	4.47 ± 0.14	14.57 ± 1.27	11.55 ± 1.25
**27**	1.89 ± 0.54	22.94 ± 1.97	> 50
**28**	4.52 ± 0.97	20.72 ± 2.04	16.37 ± 1.32
**5-FU** ^**a**^	2.20 ± 0.08	25.98 ± 1.08	38.69 ± 2.84

IC_50_ values expressed as mean ± standard deviation, n = 3

^a^Positive control

## Conclusions

A pair of new racemic mixture of xanthones, (+)-cracochinxanthone A (**1a**) and (−)-cracochinxanthone A (**1b**), along with 30 known analogues (**2**–**31**) were isolated from the stems and leaves of *C. cochinchinense.* The antiproliferative activities of some selected compounds against human HL-60, PC-3, and MDA-MB-231 cancer cell lines were screened by the trypan blue and MTT methods. The polyprenylated or geranylated xanthones exhibited potent cytotoxicity against three human malignant cell lines, which could be further developed as potential lead compounds in the design for the treatment of cancer.

## Additional files

**Additional file 1.** Minimum standards of reporting checklist.

**Additional file 2.** Supporting information.

## Abbreviations

HL-60: human myeloid leukemia cell line
PC-3: human prostate cancer cell line
MDA-MB-231: human breast carcinoma cell line
ODS: octadecyl silane
CC: column chromatography
TMS: tetramethylsilane
PE: petroleum ether
CH_2_Cl_2_: dichloromethane
EtOAc: ethyl acetate
*n*-BuOH: *n*-butyl alcohol
H_2_O: water
ECD: electronic circular dichroic spectroscopy
HPLC: high performance liquid chromatography
IC_50_: the concentration of drug required to inhibit cell growth by 50% compared with untreated control
