# Supplementary material for: A pair of new enantiomers of xanthones from the stems and leaves of Cratoxylum cochinchinense
Source: Chin Med. 2019 Mar 29;14:14. doi: 10.1186/s13020-019-0235-z (PMC6441139; doi:10.1186/s13020-019-0235-z)
Supplement: Supplementary file 2 — Additional file 2. Supporting information. [file 13020_2019_235_MOESM2_ESM.docx]

**A pair of new enantiomers of xanthones from the stems and leaves of *Cratoxylum cochinchinense***

Cuicui Jia^1,2,3*^, Chi Gong^1,3^, Hong Chen^4^, Jing Pu^1,3^, Dahong Li^1,3^, Zhanlin Li^1,3^, Huiming Hua^1,3*^

^1^ Key Laboratory of Structure-Based Drug Design & Discovery, Ministry of Education, Shenyang Pharmaceutical University, Shenyang, 110016 Liaoning, People’s Republic of China
^2^ Department of Psychiatry, Qiqihar Medical University, Qiqihar, 161006 Heilongjiang, People’s Republic of China

^3^ School of Traditional Chinese Materia Medica, Shenyang Pharmaceutical University, Shenyang, People’s Republic of China

^4^ School of Life Science and Biopharmaceutics, Shenyang Pharmaceutical University, Shenyang, People’s Republic of China

1. mail: cuicjia@163.com (C-C Jia^*^); gongchi0514@163.com (C G); chen_1992hong@163.com (H C) ; 1297688532@qq.com (J P); lidahong0203@163.com (D-H Li); lzl1030@hotmail.com (L-Z Li); huimhua@163.com (H-M Hua^*^).

*Corresponding authors:

E-mail addresses: cuicjia@163.com (C-C Jia)

Tel.: +86-0452-2663179.

E-mail addresses: huimhua@163.com (H-M Hua)

Tel.: +86-024-23986465.

**Contents**

**Figure S1**. ^1^H NMR (DMSO-*d*_6_) spectrum of compound **1**

**Figure S2**. ^13^C NMR (DMSO-*d*_6_) spectrum of compound **1**

**Figure S3**. HMQC (DMSO-*d*_6_) spectrum of compound **1**

**Figure S4**. HMBC (DMSO-*d*_6_) spectrum of compound **1**

**Figure S5**. HRESIMS (MeOH) spectrum of compound **1**

**Figure S6**. UV spectrum of compound **1**

**Figure S7**. The chiral HPLC chromatogram of **1a** and **1b**

**Table S1.** Energy analysis of **1a** at B3LYP/6-31+G(d, p) level

**Table S2.** 2D Structures of **1a** and **1b**

**Table S3.** B3LYP/6-31+G(d, p) optimized lowest energy 3D conformers of **1a**

**Figure S8.** Calculated and experimental ECD spectra of compounds **1a** and **1b**

**Spectroscopic data of known xanthones**


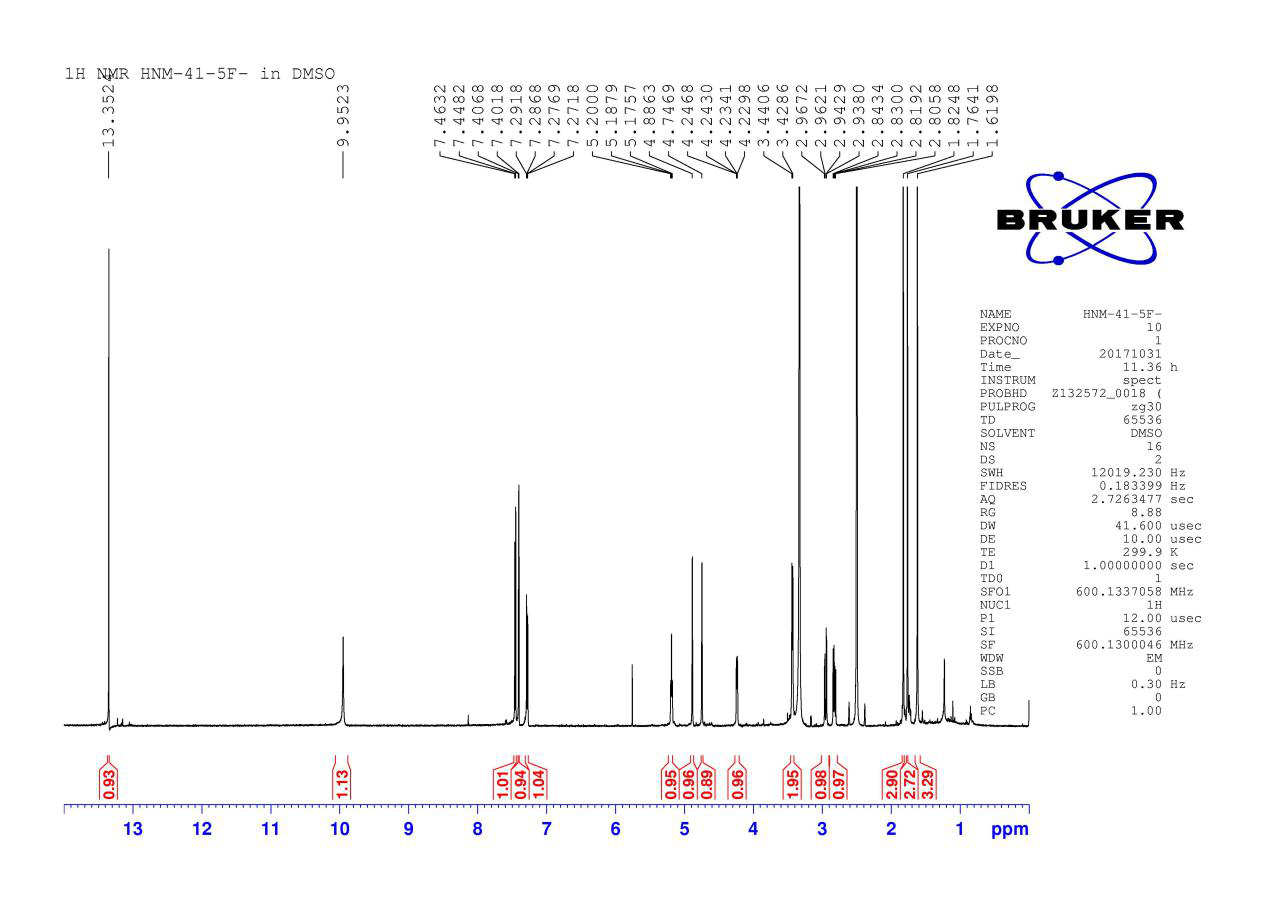


**Figure S1**. ^1^H NMR (DMSO-*d*_6_) spectrum of compound **1**

**
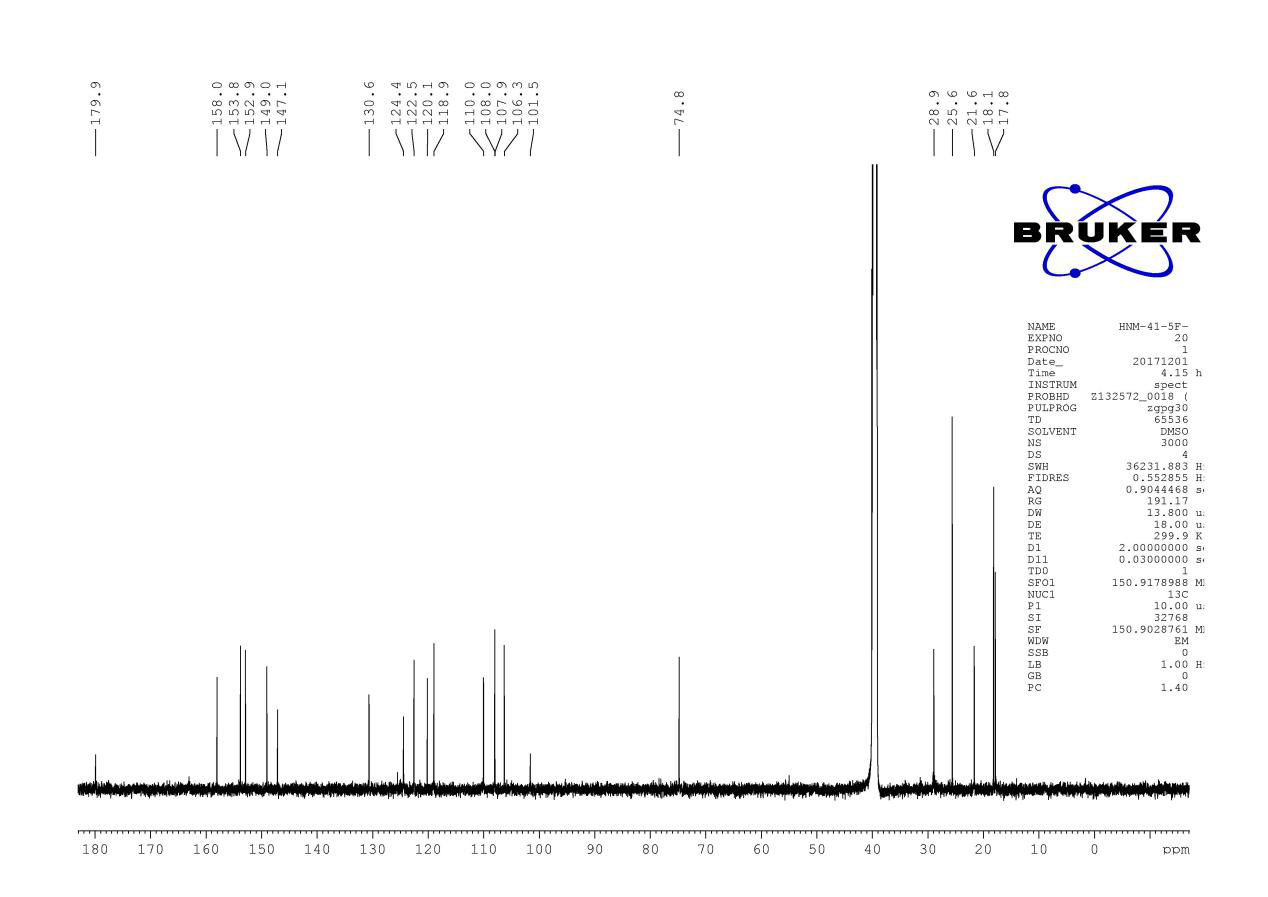
**

**Figure S2**. ^13^C NMR (DMSO-*d*_6_) spectrum of compound **1**

**
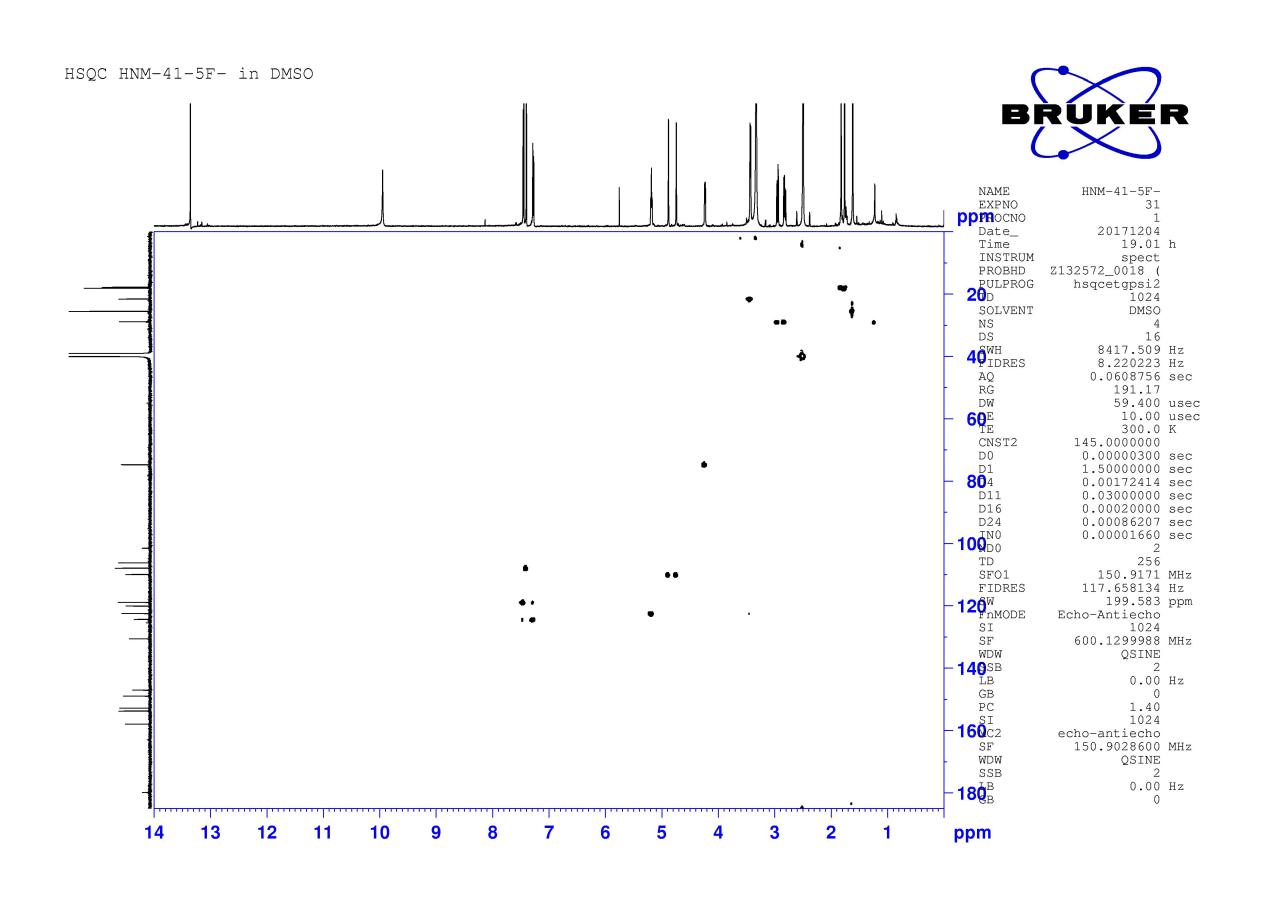
**

**Figure S3**. HMQC (DMSO-*d*_6_) spectrum of compound **1**

**
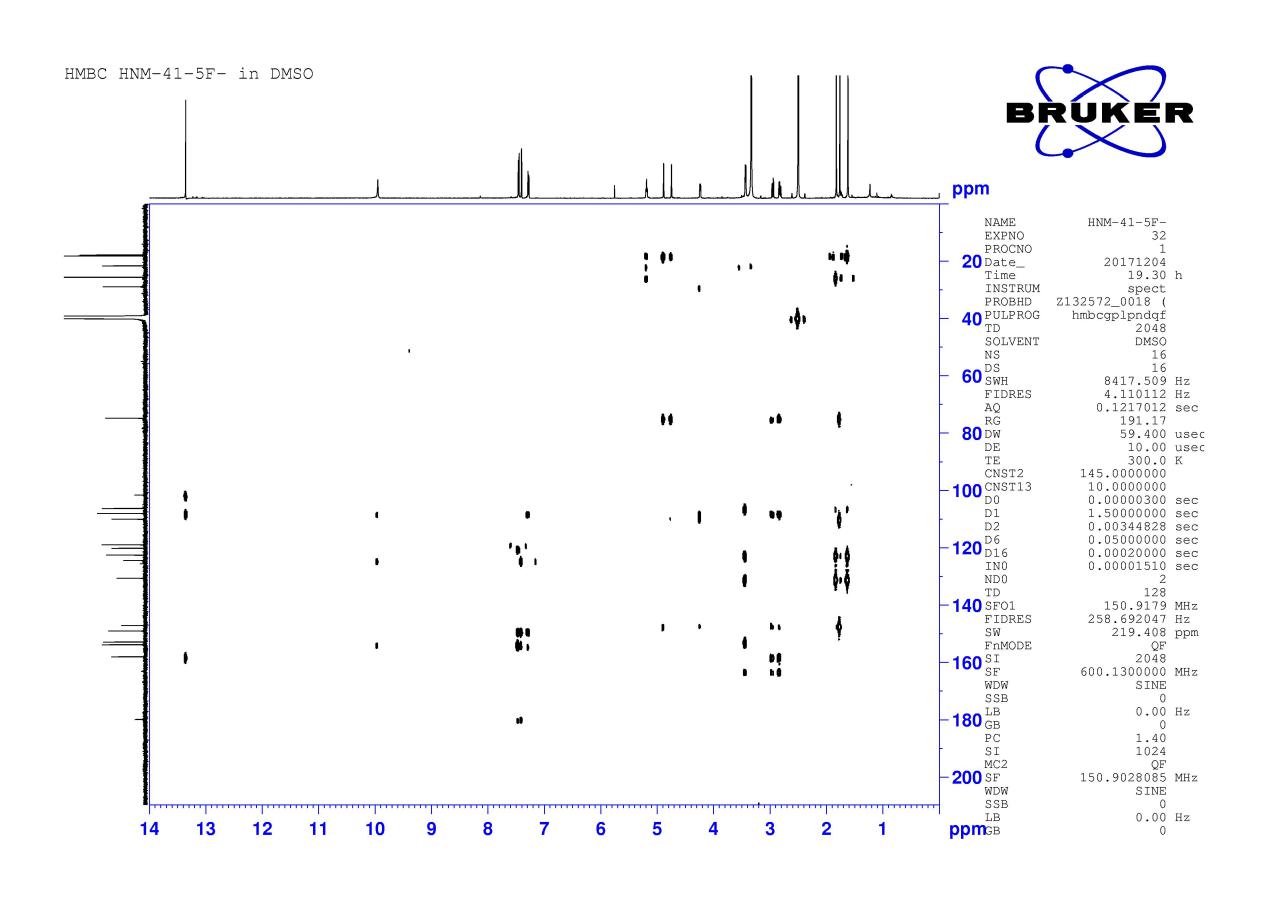
**

**Figure S4**. HMBC (DMSO-*d*_6_) spectrum of compound **1**


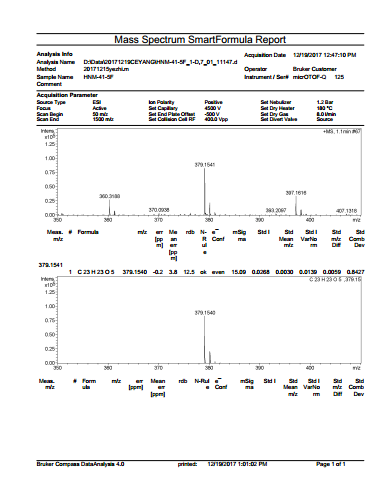


**Figure S5**. HRESIMS (MeOH) spectrum of compound **1**


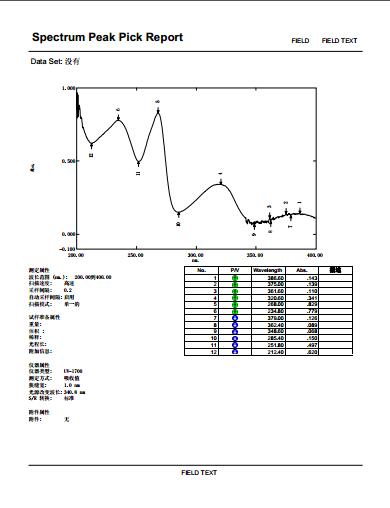


**Figure S6**. UV spectrum of compound **1**

**Figure S7**. The chiral HPLC chromatogram of **1a** and **1b**

**Computational methods**

The Spartan 14.0 (Wavefunction Inc., Irvine, CA, USA) search using molecular mechanics MMFF was performed for **1a**, which gave 100 conformers. The low-energy conformers of **1a** accounting for more than 1.0% Boltzmann distribution and less than 10.0 kJ/mol Relative Energy were further optimized in the gas phase by semi-empirical method in Gaussian 09 program package.^[1]^ Those geometeies were further optimized using the density functional theory (DFT) at the B3LYP/6-31G(d, p) level, resulted in no imaginary frequencies. Solvent effects were taken into consideration by using the polarizable continuum model (PCM). The conformers of **1a** were calculated electronic circular dichroism (ECD) by the time-dependent density functional theory (TD-DFT) method at the B3LYP/6-31G(d, p) level with the PCM model in methanol solution. The overall calculated ECD curves of **1a** were generated by Boltzmann weighting of their selected low-energy conformers using SpecDis 1.51 ^[2,3]^ with σ = 0.20 eV at-16nm shift.

**Table S1** Energy analysis of **1a** at B3LYP/6-31G(d, p) level

| Label | rel. E(kcal/mol) | | Boltzmann Dist. | |
| --- | --- | --- | --- | --- |
| **1a**-1 | | 0.05 | | 0.081 |
| **1a**-2 | | 0.47 | | 0.040 |
| **1a**-3 | | 0.49 | | 0.038 |
| **1a**-4 | | 0.01 | | 0.088 |
| **1a**-5 | | 0.01 | | 0.088 |
| **1a**-6 | | 0.10 | | 0.074 |
| **1a**-7 | | 0.01 | | 0.087 |
| **1a**-8 | | 0.01 | | 0.087 |
| **1a**-9 | | 0.10 | | 0.074 |
| **1a**-10 | | 0.43 | | 0.043 |
| **1a**-11 | | 0.38 | | 0.047 |
| **1a**-12 | | 0.05 | | 0.081 |
| **1a**-13 | | 0.43 | | 0.043 |
| **1a**-14 | | 0.47 | | 0.040 |
| **1a**-15 | | 1.00 | | 0.089 |

**References**

[1] Frisch M. J., Trucks G. W., Schlegel H. B., Scuseria G. E., Robb M. A., Cheeseman J. R., Scalmani G., Barone V., Mennucci B., Petersson G. A., Nakatsuji H., Caricato M., Li X., Hratchian H. P., Izmaylov A. F., Bloino, J., Zheng, G., Sonnenberg, J. L., Hada, M., Ehara, M., Toyota, K., Fukuda, R., Hasegawa, J., Ishida, M., Nakajima, T., Honda, Y., Kitao, O., Nakai, H., Vreven, T., Montgomery, J. A., Jr., Peralta, J. E., Ogliaro, F., Bearpark M., Heyd J. J., Brothers E., Kudin K. N., Staroverov V. N., Kobayashi R., Normand J., Raghavachari K., Rendell A., Burant J. C., Iyengar S. S., Tomasi J., Cossi M., Rega N., Millam J. M., Klene M., Knox J. E., Cross J. B., Bakken V., Adamo C., Jaramillo J., Gomperts R., Stratmann R. E., Yazyev O., Austin A. J., Cammi R., Pomelli C., Ochterski J. W., Martin R. L., Morokuma K., Zarzewski V. G., Voth G. A., Salvador P ., Dannenberg J.J., Dapprich S., Daniels A.D., Farkas O., Foresman J. B., Ortiz J. V., Cioslowski J., Fox D. J. Gaussian 09, Revision C1, Gaussian, Inc.: Wallingford, CT, 2010.

[2]. Bruhn T., Hemberger Y., Schaumlöffel A., Bringmann G. *Spec Dis*, version 1.51, University of Würzburg, Germany, 2010.

[3] Bruhn T., Schaumlöffel A., Hemberger Y., Bringmann G. Quantifying the Comparison of Calculated and Experimental Electronic Circular Dichroism Spectra, Chirality 2013, 25, 243–249.

**Table S2** 2D Structures of **1a** and **1b**

| label | structure |
| --- | --- |
| **1a** |  |
| **1b** |  |

**Table S3.** B3LYP/6-31G(d, p) optimized lowest energy 3D conformers of **1a**

| label | conformer | Boltzmann weighting factors |
| --- | --- | --- |
| **1a**-1 | 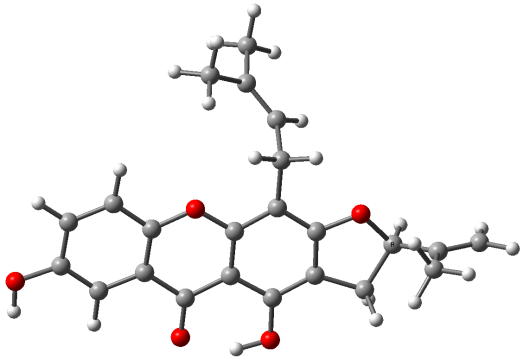 | 8.06 |
| **1a**-2 | 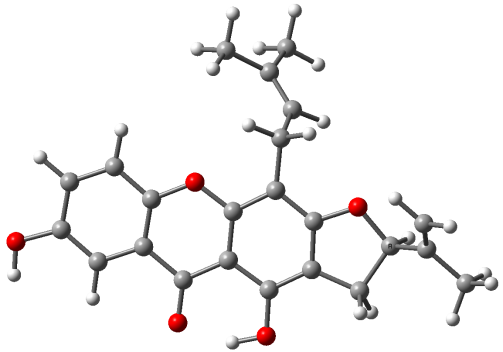 | 3.98 |
| **1a**-3 | 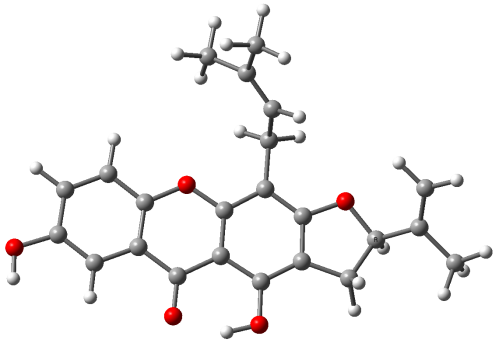 | 3.85 |
| **1a**-4 | 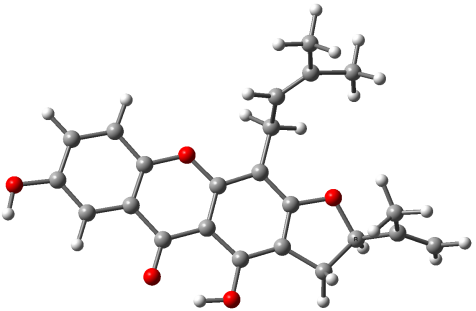 | 8.84 |
| **1a**-5 | 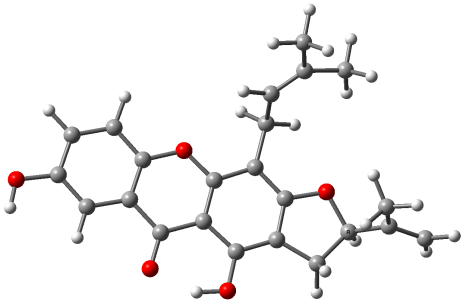 | 8.84 |
| **1a**-6 | 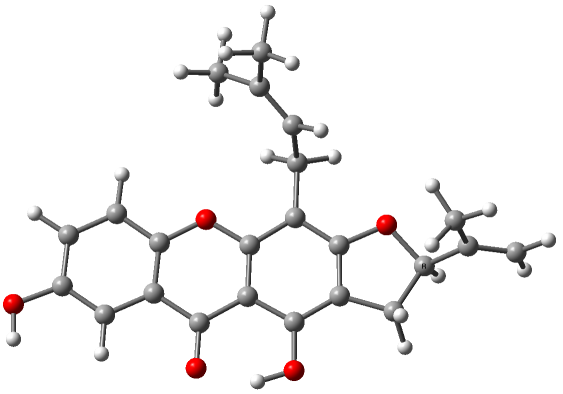 | 7.40 |
| **1a**-7 | 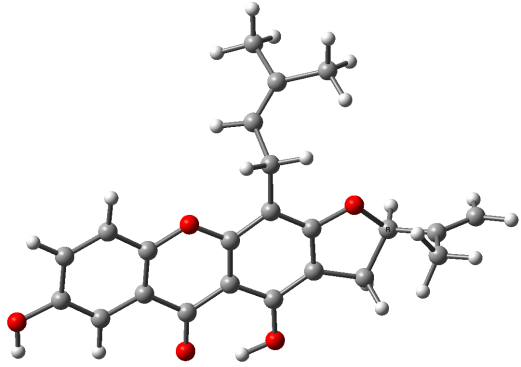 | 4.29 |
| **1a**-8 | 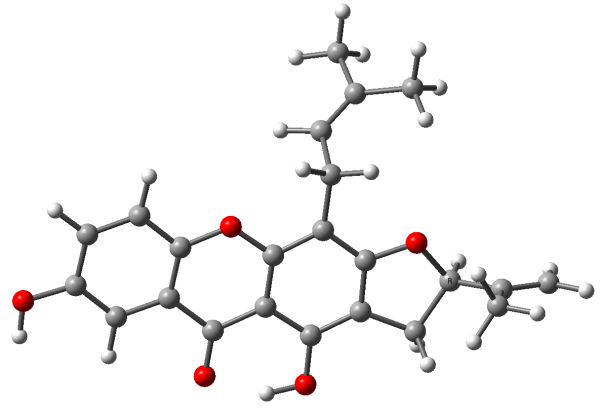 | 4.64 |
| **1a**-9 | 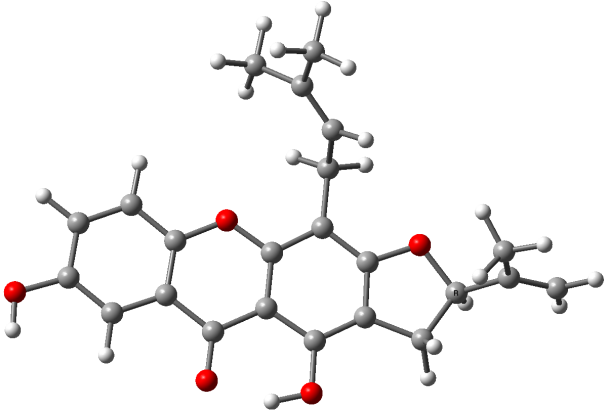 | 8.06 |
| **1a**-10 | 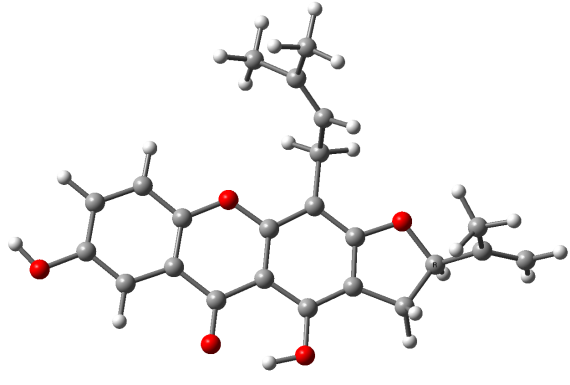 | 4.29 |
| **1a**-11 | 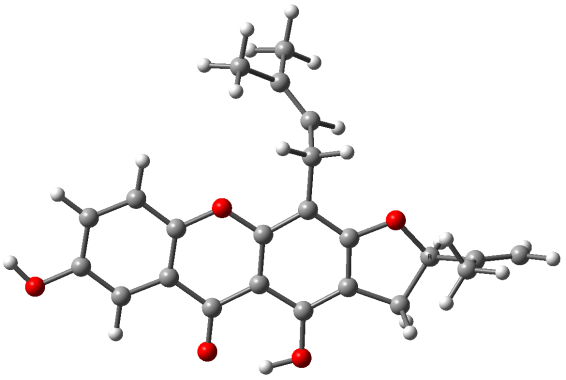 | 4.64 |
| **1a**-12 | 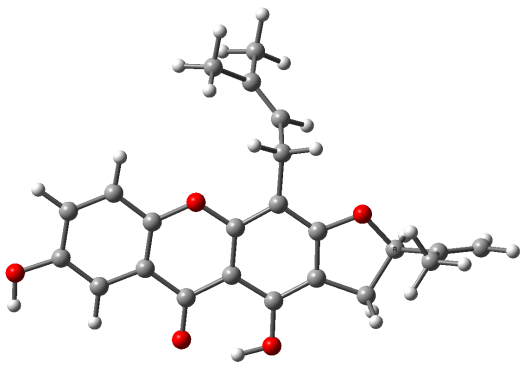 | 8.06 |
| **1a**-13 | 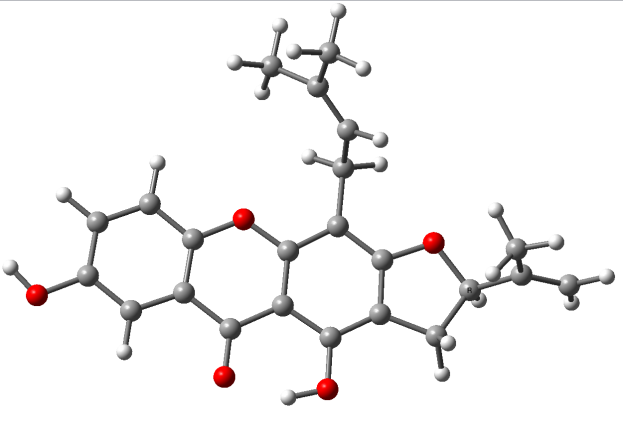 | 4.29 |
| **1a**-14 | 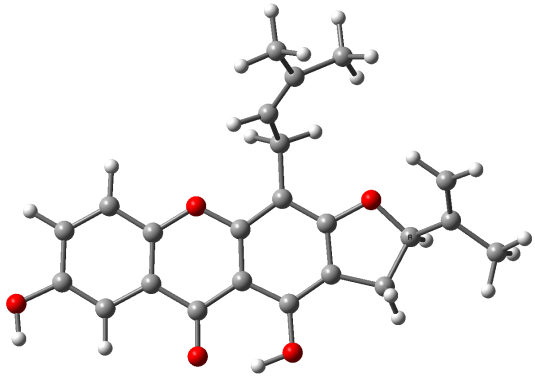 | 4.02 |
| **1a**-15 | 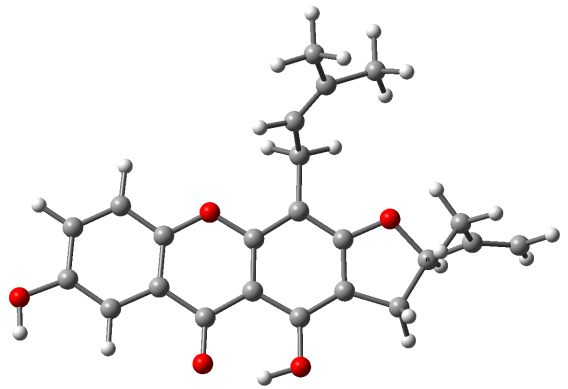 | 8.84 |

**
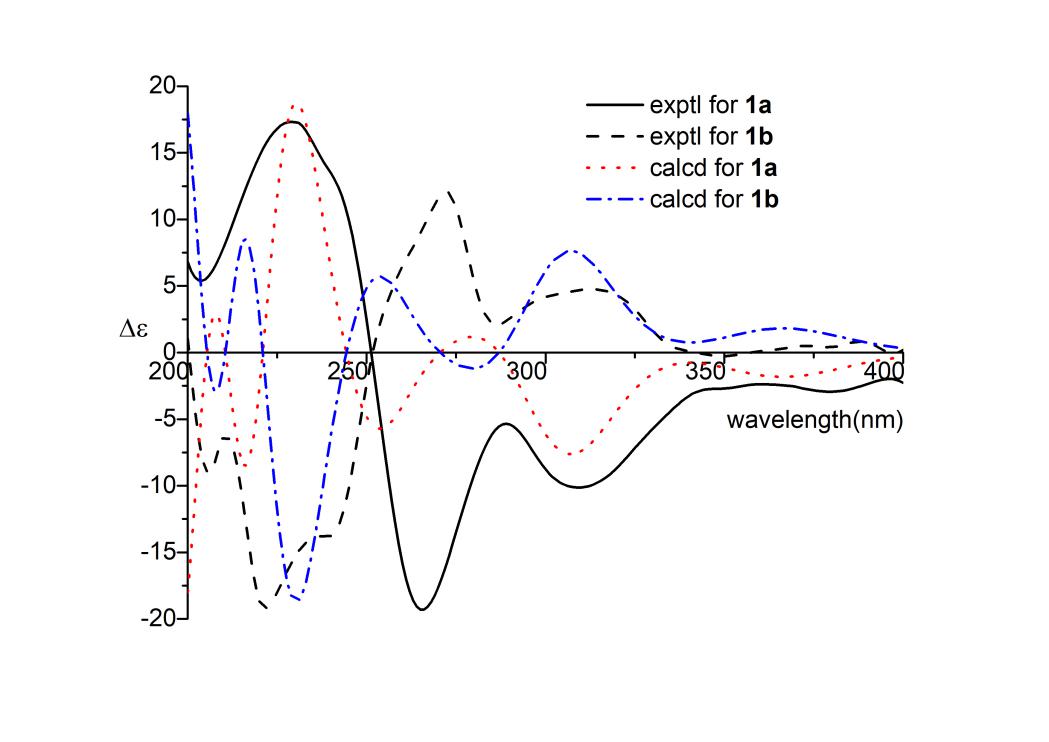
**

**Figure S8.** Calculated and experimental ECD spectra of **1**

**Spectroscopic data of known xanthones**

Cochinchinoxanthone (**2**)

^1^H NMR (600 MHz, DMSO-*d*_6_): *δ*_H_ 12.55 (1H, s, 1-OH), 10.98 (1H, brs, 3-OH), 7.41 (1H, d, *J* = 7.0 Hz, H-8), 5.92 (1H, d, *J* = 2.0 Hz, H-2), 5.95 (1H, d, *J* = 2.0 Hz, H-4), 4.33 (1H, m), 1.57 (3H, s, CH_3_-15), 1.31 (3H, s, CH_3_-14), 1.31 (3H, s, CH_3_-19), 1.04 (3H, s, CH_3_-20).

^13^C NMR (150 MHz, DMSO-*d*_6_): *δ*_C_ 203.1 (C-6), 178.8 (C-9), 167.5 (C-1), 164.3 (C-3), 160.9 (C-4a), 134.6 (C-8a), 133.8 (C-18), 132.8 (C-8), 118.4 (C-17), 99.7 (C-9a), 96.1 (C-2), 94.9 (C-4), 89.8 (C-10a), 83.7 (C-13), 83.1 (C-5), 47.9 (C-12), 46.4 (C-7), 30.0 (C-15), 28.8 (C-14), 28.7 (C-16), 25.4 (C-11), 24.6 (C-19), 16.6 (C-20).

1,4,7-Trihydroxy-8-methoxyxanthone (**3**)

^1^H NMR (600 MHz, DMSO-*d*_6_): *δ*_H_ 12.22 (1H, s, OH-1), 9.61 (1H, s), 9.53 (1H, s), 7.44 (1H, d, *J* = 9.1 Hz, H-6), 7.29 (1H, d, *J* = 9.1 Hz, H-5), 7.20 (1H, d, *J* = 8.7 Hz, H-2), 6.58 (1H, d, *J* = 8.7 Hz, H-3), 3.82 (3H, s, 8-OCH_3_).

^13^C NMR (150 MHz, DMSO-*d*_6_): *δ*_C_ 182.0 (C-9), 152.5 (C-1), 149.6 (C-10a), 147.8 (C-7), 145.1 (C-8), 143.4 (C-4a), 136.8 (C-4), 125.2 (C-6), 122.8 (C-3), 115.6 (C-8a), 113.6 (C-5), 108.9 (C-9a), 108.3 (C-2), 61.0 (8-OCH3).

entisein (**4**)

^1^H NMR (600 MHz, DMSO-*d*_6_): *δ*_H_ 12.90 (1H, s, OH-1), 7.47 (1H, d, *J* = 9.1 Hz, H-5), 7.40 (1H, d, *J* = 3.1 Hz, H-8), 7.28 (1H, dd, *J* = 9.1, 3.1 Hz, H-6), 6.36 (1H, d, *J* = 2.1 Hz, H-4), 6.18 (1H, d, *J* = 2.1 Hz, H-2).

^13^C NMR (150 MHz, DMSO-*d*_6_): *δ*_C_ 179.7 (C-9), 165.7 (C-3), 162.8 (C-1), 157.5 (C-4a), 153.9 (C-7), 149.0 (C-10a), 124.5 (C-6), 120.4 (C-8a), 119 (C-5), 108 (C-8), 101.9 (C-9a), 97.9 (C-2), 93.8 (C-4).

1,6-Dihydroxy-2,5,8-trimethoxyxanthone (**5**)

^1^H NMR (600 MHz, DMSO-*d*_6_): *δ*_H_ 13.36 (1H, s, OH-1), 7.43 (1H, d, *J* = 9.0 Hz, H-3), 6.90 (1H, d, *J* = 9.0 Hz, H-4), 6.70 (1H, s, H-7), 3.86 (3H, s, 8-OCH_3_), 3.81 (3H, s, 2-OCH_3_), 3.78 (3H, s, 6-OCH_3_).

^13^C NMR (150 MHz, DMSO-*d*_6_): *δ*_C_ 181 (C-9), 158.9 (C-10a), 154.2 (C-6), 153.3 (C-8), 150.2 (C-1), 148.4 (C-4a), 142.2 (C-2), 138.7 (C-5), 120.5 (C-3), 108.2 (C-9a), 106.9 (C-8a), 104.7 (C-4), 99.2 (C-7), 61.7 (8-OCH_3_), 60.9 (5-OCH_3_), 56.6 (2-OCH_3_).

1,7-Dihydroxyxanthone (**6**)

^1^H NMR (600 MHz, DMSO-*d*_6_): *δ*_H_ 12.65 (1H, s, OH-1), 10.11 (1H, s, OH-7), 7.56 (1H, d, *J* = 9.1 Hz, H-5), 7.46 (1H, d, *J* = 3.0 Hz, H-8), 7.33 (1H, dd, *J* = 9.1, 3.0 Hz, H-6), 7.72 (1H, t, *J* = 8.3 Hz, H-3), 7.06 (1H, d, *J* = 8.3 Hz, H-4), 6.80 (1H, d, *J* = 8.3 Hz, H-2).

^13^C NMR (150 MHz, DMSO-*d*_6_): *δ*_C_ 181.6 (C-9), 160.9 (C-1), 155.9 (C-4a), 154.2 (C-7), 149.4 (C-10a), 137.2 (C-3), 125.6 (C-6), 120.4 (C-8a), 119.4 (C-5), 109.7 (C-2), 107.9 (C-8), 107.8 (C-9a), 107.2 (C-4).

1,7-Dihydroxy-4-methoxyxanthone (**7**)

^1^H NMR (600 MHz, DMSO-*d*_6_): *δ*_H_ 12.00 (1H, s, OH-1), 7.47 (1H, d, *J* = 8.8 Hz, H-2), 6.72 (1H, d, *J* = 8.8 Hz, H-3), 7.62 (1H, d, *J* = 9.1 Hz, H-5), 7.37 (1H, dd, *J* = 9.1, 2.9 Hz, H-6), 7.46 (1H, d, *J* = 2.9 Hz, H-8), 3.90 (3H, s, 4-OCH_3_).

^13^C NMR (150 MHz, DMSO-*d*_6_): *δ*_C_ 181.6 (C-9), 154.2 (C-1), 153.3 (C-7), 149.3 (C-4a), 145.2 (C-10a), 139.8 (C-4), 125.7 (C-6), 120.5 (C-8a), 120.1 (C-3), 119.7 (C-5), 108.4 (C-9a), 107.9 (C-8), 107.7 (C-2), 57.6 (4-OCH_3_).

1,7-Dihydroxy-3,6-dimethoxyxanthone (**8**)

^1^H NMR (600 MHz, DMSO-*d*_6_): *δ*_H_ 13.16 (1H, s, OH-1), 6.60 (1H, d, *J* = 1.9 Hz, H-2), 6.36 (1H, d, *J* = 1.9 Hz, H-4), 7.44 (1H, s, H-8), 6.91 (1H, s, H-5), 3.89 (3H, s), 3.86 (3H, s).

^13^C NMR (150 MHz, DMSO-*d*_6_): *δ*_C_ 179 (C-9), 165.7 (C-3), 162.3 (C-1), 157.1 (C-4a), 155.2 (C-6), 152.1 (C-10a), 146.4 (C-7), 111.3 (C-8a), 104.6 (C-8), 102.7 (C-9a), 102.5 (C-5), 96.9 (C-2), 92.5 (C-4), 56.1 (6-OCH_3_), 55.9 (3-OCH_3_).

1,7-Dihydroxy-8-methoxyxanthone (**9**)

^1^H NMR (600 MHz, DMSO-*d*_6_): *δ*_H_ 13.03 (1H, s, OH-1), 9.87 (1H, s, OH-7), 7.67 (1H, t, *J* = 8.3 Hz, H-3), 6.97 (1H, d, *J* = 8.3 Hz, H-4), 6.75 (1H, d, *J* = 8.3 Hz, H-2), 7.47 (1H, d, *J* = 9.0 Hz, H-6), 7.28 (1H, d, *J* = 9.0 Hz, H-5), 3.83 (3H, s, 8-OCH_3_).

^13^C NMR (150 MHz, DMSO-*d*_6_): *δ*_C_ 182 (C-9), 161.2 (C-1), 155.2 (C-4a), 149.5 (C-8), 147.1 (C-10a), 145.2 (C-7), 136.9 (C-3), 125.2 (C-6), 115.2 (C-8a), 113.4 (C-2), 109.7 (C-4), 108.4 (C-5), 101.5 (C-9a), 61 (8-OCH_3_).

1,5,6-Trihydroxy-7-methoxyxanthone (**10**)

^1^H NMR (600 MHz, DMSO-*d*_6_): *δ*_H_ 12.99 (1H, s, OH-1), 8.13 (1H, s), 9.96 (1H, s), 7.67 (1H, t, *J* = 8.2 Hz, H-3), 7.06 (1H, dd, *J* = 8.2, 0.8 Hz, H-4), 6.77 (1H, dd, *J* = 8.2, 0.8 Hz, H-2), 7.12 (1H, s), 3.91 (3H, s, 7-OCH_3_).

^13^C NMR (150 MHz, DMSO-*d*_6_): *δ*_C_ 180.7 (C-9), 160.9 (C-1), 155.7 (C-4a), 146.2 (C-7), 142.9 (C-6), 142.6 (C-10a), 136.4 (C-3), 133.3 (C-5), 111.4 (C-8a), 109.6 (C-2), 107.7 (C-9a), 107.1 (C-4), 95.3 (C-8), 56.0 (7-OCH_3_).

1,4,7-Trihydroxyxanthone (**11**)

^1^H NMR (600 MHz, DMSO-*d*_6_): *δ*_H_ 11.90 (1H, s, OH-1), 7.56 (1H, d, *J* = 3.0 Hz, H-8), 7.46 (1H, d, *J* = 8.9 Hz, H-5), 7.37 (1H, dd, *J* = 8.9, 2.9 Hz, H-6), 7.26 (1H, d, *J* = 8.8 Hz, H-2), 6.61 (1H, d, *J* = 8.8 Hz, H-3).

1,5,6-Trihydroxy-3,7-dimethoxyxanthone (**12**)

^1^H NMR (600 MHz, DMSO-*d*_6_): *δ*_H_ 13.18 (1H, s, OH-1), 10.05 (1H, s), 9.70 (1H, s), 7.09 (1H, s, H-8), 6.59 (1H, d, *J* = 2.2 Hz, H-4), 6.36 (1H, d, *J* = 2.2 Hz, H-2), 3.89 (3H, s, 3-OCH_3_), 3.88 (3H, s, 7-OCH_3_).

^13^C NMR (150 MHz, DMSO-*d*_6_): *δ*_C_ 179.5 (C-9), 165.7 (C-3), 162.4 (C-1), 157.1 (C-4a), 146 (C-7), 142.3 (C-6), 142.3 (C-10a), 133.4 (C-5), 111.2 (C-8a), 102.5 (C-9a), 96.8 (C-2), 95.3 (C-8), 92.4 (C-4), 56 (3 and 7-OCH_3_).

1,3,5,6-Tetrahydroxyxanthone (**13**)

^1^H NMR (600 MHz, DMSO-*d*_6_): *δ*_H_ 13.12 (1H, s, OH-1), 7.49 and 6.92 (each 1H, d, *J* = 8.7 Hz, H-8, H-7), 6.39 and 6.16 (each 1H, d, *J* = 2.0 Hz, H-4, H-2).

1,3,6,7-Tetrahydroxyxanthone (**14**)

^1^H NMR (600 MHz, DMSO-*d*_6_): *δ*_H_ 13.16 (1H, s, OH-1), 7.36 (1H, s, H-8), 6.85 (1H, s, H-5), 6.31 (1H, d, *J* = 2.0 Hz, H-4), 6.14 (1H, d, *J* = 2.0 Hz, H-2).

1,3,6-Trihydroxy-7-methoxyxanthone (**15**)

^1^H NMR (600 MHz, DMSO-*d*_6_): *δ*_H_ 13.13 (1H, s, OH-1), 7.43 (1H, s, H-8), 6.91 (1H, s, H-5), 6.35 (1H, d, *J* = 1.7 Hz, H-2), 6.17 (1H, d, *J* = 1.7 Hz, H-4), 3.88 (3H, s, CH_3_-7).

^13^C NMR (150 MHz, DMSO-*d*_6_): *δ*_C_ 178.8 (C-9), 164.8 (C-3), 162.6 (C-1), 157.3 (C-4a), 154.7 (C-6), 151.9 (C-10a), 146.1 (C-7), 111.5 (C-8a), 104.7 (C-8), 102.8 (C-5), 101.6 (C-9a), 97.9 (C-2), 93.7 (C-4), 55.9 (7-OCH_3_).

Cratoxanthone C (**16**)

^1^H NMR (600 MHz, DMSO-*d*_6_): *δ*_H_ 13.17 (1H, s, 1-OH), 8.33 (1H, s, 6-OH), 7.27 (1H, d, *J* = 9.0 Hz, H-3), 6.74 (1H, d, *J* = 9.0 Hz, H-4), 6.19 (1H, s), 3.77 (3H, s, 2-OCH_3_), 3.72 (3H, s, 7-OCH_3_), 3.78 (3H, s, 8-OCH_3_).

^13^C NMR (150 MHz, DMSO-*d*_6_): *δ*_C_ 178.7 (C-9), 155.7 (C-6), 155.3 (C-10a), 152.1 (C-8), 150.6 (C-1), 148.3 (C-4a), 141.8 (C-2), 141.1 (C-7), 119.3 (C-3), 108 (C-9a), 103.8 (C-8a), 101.8 (C-4), 100 (C-5), 61.2 (7-OCH_3_), 59.8 (8-OCH_3_), 56.6 (2-OCH_3_).

1,2,4-Trimethoxy-3,8-dimethoxyxanthone (**17**)

^1^H NMR (600 MHz, DMSO-*d*_6_): *δ*_H_ 13.21 (1H, s, 8-OH), 10.81 (1H, s, 3-OH), 7.64 (1H, t, *J* = 8.2 Hz, H-6), 7.02 (1H, d, *J* = 8.2 Hz, H-5), 6.76 (1H, d, *J* = 8.2 Hz, H-7), 3.81 (3H, s, 1-OCH_3_), 3.83 (3H, s, 2-OCH_3_), 3.87 (3H, s, 4-OCH_3_).

^13^C NMR (150 MHz, DMSO-*d*_6_): *δ*_C_ 180.7 (C-9), 161.2 (C-8), 154.8 (C-10a), 150.9 (C-3), 148.7 (C-2), 147.4 (C-4a), 139.1 (C-4), 136.4 (C-6), 132 (C-1), 115.2 (C-5), 110.2 (C-9a), 108 (C-8a), 106.6 (C-7), 61.7 (1-OCH_3_), 61.1 (2 and 4-OCH_3_).

1,3,7-Trihydroxy-2-(3-methylbut-2-enyl)-xanthone (**18**)

^1^H NMR (600 MHz, DMSO-*d*_6_): *δ*_H_ 13.16 (1H, s, OH-1), 11.03 (1H, s, OH-7), 10.00 (1H, s, OH-3), 6.43 (1H, s, H-4), 7.46 (1H, d, *J* = 9.0 Hz, H-5), 7.41 (1H, d, *J* = 3.0 Hz, H-8), 7.27 (1H, dd, *J* = 9.0, 3.0 Hz, H-6), 3.23 (2H, d, *J* = 7.1 Hz, H-1′), 5.18 (1H, t, *J* = 7.1 Hz, H-2′), 1.74 (3H, s, CH_3_-4′), 1.62 (3H, s, CH_3_-5′).

^13^C NMR (150 MHz, DMSO-*d*_6_): *δ*_C_ 179.8 (C-9), 163.3 (C-3), 159.5 (C-1), 155.3 (C-4a), 153.8 (C-7), 148.9 (C-10a), 130.7 (C-3'), 124.3 (C-2'), 122.2 (C-6), 120.4 (C-8a), 119 (C-5), 109.8 (C-2), 108 (C-8), 101.7 (C-9a), 93 (C-4), 25.5 (C-5'), 20.9 (C-1'), 17.7 (C-4').

Dulcisxanthone B (**19**)

^1^H NMR (600 MHz, DMSO-*d*_6_): *δ*_H_ 13.77 (1H, s, OH-1), 6.78 (1H, s, H-5), 6.57 (1H, s, H-4), 4.03 (2H, d, *J* = 6.9 Hz, H-1''), 5.19 (1H, t, *J* = 6.9 Hz, H-2''), 1.77 (3H, s, CH_3_-4''), 1.61 (3H, s, CH_3_-5''), 3.22 (2H, d, *J* = 7.2 Hz, H-1'), 5.13 (1H, t, *J* = 7.2 Hz, H-2'), 1.72 (3H, s, CH_3_-4'), 1.61 (3H, s, CH_3_-5'), 3.88 (3H, s, OCH_3_-3).

^13^C NMR (150 MHz, DMSO-*d*_6_): *δ*_C_ 181.7 (C-9), 162.9 (C-3), 158.8 (C-1), 154.7 (C-4a), 152.7 (C-6), 152.1 (C-10a), 141.1 (C-7), 130.7 (C-3'), 130.2 (C-3''), 127.6 (C-8), 123.7 (C-2''), 122.3 (C-2'), 110.2 (C-8a), 110 (C-2), 102.8 (C-9a), 100.1 (C-5), 89.3 (C-4), 56.2 (3-OCH_3_), 25.6 (C-1''), 25.5 (C-5'), 25.5 (C-5''), 20.9 (C-1'), 18.1 (C-4''), 17.6 (C-4').

Cudratricusxanthone E (**20**)

^1^H NMR (600 MHz, DMSO-*d*_6_): *δ*_H_ 13.38 (1H, s, OH-1), 10.67 (1H, s), 9.70 (1H, s), 7.37 (1H, s, H-8), 6.86 (1H, s, H-5), 3.46 (2H, d, *J* = 7.2 Hz, H-1''), 5.14 (1H, t, *J* = 7.2 Hz, H-2''), 1.85 (3H, s, CH_3_-4''), 1.63 (3H, s, CH_3_-5''), 3.21 (2H, d, *J* = 6.9 Hz, H-1'), 5.14 (1H, t, *J* = 6.9 Hz, H-2'), 1.73 (3H, s, CH_3_-4'), 1.62 (3H, s, CH_3_-5').

^13^C NMR (150 MHz, DMSO-*d*_6_): *δ*_C_ 179.3 (C-9), 159.5 (C-3), 157.3 (C-1), 154 (C-6), 152.4 (C-4a), 150.7 (C-10a), 143.7 (C-7), 130.6 (C-3'), 130.6 (C-3''), 122.6 (C-2'), 122.5 (C-2''), 111.6 (C-8a), 110 (C-2), 108 (C-8), 105.8 (C-4), 102.5 (C-5), 101.7 (C-9a), 25.6 (C-5'), 25.6 (C-5''), 21.6 (C-1''), 21.3 (C-1'), 17.8 (C-4' and 4'').

γ-Mangostin (**21**)

^1^H NMR (600 MHz, DMSO-*d*_6_): *δ*_H_ 14.07 (1H, s, OH-1), 6.60 (1H, s, H-5), 6.54 (1H, s, H-4), 3.22 (2H, d, *J* = 6.9 Hz, H-1'), 5.13 (1H, t, *J* = 6.9 Hz, H-2'), 1.72 (3H, s, CH_3_-4'), 1.61 (3H, s, CH_3_-5'), 4.00 (2H, d, *J* = 6.6 Hz, H-1''), 5.22 (1H, t, *J* = 6.6 Hz, H-2''), 1.76 (3H, s, CH_3_-4''), 1.61 (3H, s, CH_3_-5'').

^13^C NMR (150 MHz, DMSO-*d*_6_): *δ*_C_ 181.2 (C-9), 162.6 (C-3), 158.8 (C-1), 155.6 (C-4a), 154.6 (C-10a), 152.8 (C-6), 141.9 (C-7), 130.6 (C-3'), 129.9 (C-3''), 127.9 (C-8), 123.9 (C-2'), 122.4 (C-2''), 109.7 (C-8a), 108.6 (C-2), 103.9 (C-9a), 102.7 (C-5), 99.6 (C-4), 25.7 (C-5'), 25.5 (C-5''), 25.4 (C-1'), 20.9 (C-1''), 18.1 (C-4'), 17.2 (C-4'').

1,3,7-Trihydroxy-2,4-diisoprenylxanthone (**22**)

^1^H NMR (600 MHz, DMSO-*d*_6_): *δ*_H_ 13.15 (1H, s, OH-1), 7.44 (1H, d, *J* = 9.0 Hz, H-5), 7.41 (1H, d, *J* = 3.0 Hz, H-8), 7.29 (1H, dd, *J* = 9.0, 3.0 Hz, H-6), 5.15 (1H, t, *J* = 7.1 Hz, H-2′), 5.14 (1H, t, *J* = 6.1 Hz, H-2′′), 3.48 (2H, d, *J* = 7.1 Hz, H-1′), 3.33 (2H, d, *J* = 6.1 Hz, H-1′′), 1.83 (3H, s, CH_3_-4′), 1.74 (3H, s, CH_3_-4′′), 1.62 (6H, s, CH_3_-5′, 5′′).

Cochinchinone A (**23**)

^1^H NMR (600 MHz, DMSO-*d*_6_): *δ*_H_ 13.13 (1H, s, OH-1), 9.92 (2H, s, OH-3, 7), 7.40 (1H, d, *J* = 2.9 Hz, H-8), 7.38 (1H, d, *J* = 8.9 Hz, H-5), 7.27 (1H, dd, *J* = 8.9, 2.9 Hz, H-6), 3.32 (2H, d, *J* = 6.8 Hz, H-1'), 5.15 (1H, t, *J* = 6.8 Hz, H-2'), 1.73 (3H, s,CH_3_-4'), 1.62 (3H, s, CH_3_-5'), 3.46 (2H, d, *J* = 7.0 Hz, H-1''), 5.13 (1H, t, *J* = 7.0 Hz, H-2''), 1.90 (2H, m, H-4''), 1.95 (2H, m, H-5''), 4.92 (1H, t, *J* = 6.3 Hz, H-6''), 1.41 (3H, s, CH_3_-8''), 1.82 (3H, s, CH_3_-9''), 1.43 (3H, s, CH_3_-10'').

^13^C NMR (150 MHz, DMSO-*d*_6_): *δ*_C_ 180.1 (C-9), 160.5 (C-3), 157.5 (C-1), 153.8 (C-7), 152.6 (C-4a), 149.1 (C-10a), 134.1 (C-3''), 130.7 (C-3'), 130.5 (C-7''), 124.4 (C-6), 123.9 (C-6''), 122.5 (C-2'), 122.4 (C-2''), 120.1 (C-8a), 118.8 (C-5), 110.1 (C-2), 107.9 (C-8), 106 (C-4), 102.1 (C-9a), 39.1 (C-4''), 26 (C-5''), 25.5 (C-5'), 25.3 (C-8''), 21.5 (C-1''), 21.3 (C-1'), 17.8 (C-4'), 17.4 (C-10''), 16 (C-9'').

Cochinchinone B (**24**)

^1^H NMR (600 MHz, DMSO-*d*_6_): *δ*_H_ 13.79 (1H, s, OH-1), 7.17 (1H, s, H-8), 6.38 (1H, s, H-4), 3.20 (2H, d, *J* = 7.2 Hz, H-1'), 5.17 (1H, t, *J* = 7.2 Hz, H-2'), 1.72 (3H, s,CH_3_-4'), 1.62 (3H, s, CH_3_-5'), 3.44 (2H, d, *J* = 7.1 Hz, H-1''), 5.19 (1H, t, *J* = 7.1 Hz, H-2''), 1.98 (2H, m, H-4''), 1.90 (2H, d, *J* = 6.9 Hz, H-5''), 4.97 (1H, t, *J* = 6.9 Hz, H-6''), 1.45 (3H, s, CH_3_-8''), 1.84 (3H, s, CH_3_-9'') 1.48 (3H, s, CH_3_-10'').

^13^C NMR (150 MHz, DMSO-*d*_6_): *δ*_C_ 178.8 (C-9), 164.4 (C-3), 162.2 (C-1), 159.3 (C-4a), 154.9 (C-10a), 149.5 (C-6), 143.4 (C-7), 133.8 (C-3''), 130.6 (C-3'), 130.6 (C-7''), 124 (C-6''), 123.9 (C-2'), 122.7 (C-2''), 110.3 (C-5), 110.1 (C-8a), 109.3 (C-2), 104.1 (C-8), 101.1 (C-9a), 92.8 (C-4), 39.4 (C-5''), 26.1 (C-4''), 25.5 (C-5'), 25.5 (C-8''), 22.2 (C-1''), 21 (C-1'), 17.8 (C-4'), 17.5 (C-10''), 16 (C-9'').

Pruniflorone Q (**25**)

^1^H NMR (600 MHz, DMSO-*d*_6_): *δ*_H_ 13.79 (1H, s, OH-1), 7.29 (1H, s, H-8), 6.73 (1H, s, H-5), 3.29 (2H, d, *J* = 6.8 Hz, H-1'), 5.12 (1H, t, *J* = 6.8 Hz, H-2'), 1.73 (3H, s,CH_3_-4'), 1.62 (3H, s, CH_3_-5'), 3.44 (2H, d, *J* = 7.1 Hz, H-1''), 5.15 (1H, t, *J* = 7.1 Hz, H-2''), 1.95 (2H, m, H-4''), 1.88 (2H, d, *J* = 6.8 Hz, H-5''), 4.93 (1H, t, *J* = 6.8 Hz, H-6''), 1.45(3H, s, CH_3_-8''), 1.80 (3H, s, CH_3_-9''), 1.43 (3H, s, CH_3_-10'').

^13^C NMR (150 MHz, DMSO-*d*_6_): *δ*_C_ 179.1 (C-9), 159.5 (C-3), 157.3 (C-1), 155.2 (C-4a), 152.5 (C-6), 151.3 (C-10a), 144 (C-7), 130.9 (C-3''), 130.6 (C-7''), 130.5 (C-3'), 123.9 (C-6''), 122.7 (C-2'), 122.5 (C-2''), 110.9 (C-8a), 109.9 (C-8), 107.4 (C-2), 105.8 (C-4), 102.2 (C-5), 101.6 (C-9a), 39.4 (C-4''), 26 (C-5''), 25.5 (C-5'), 25.3 (C-10''), 21.6 (C-1''), 21.3 (C-1'), 17.8 (C-4'), 17.4 (C-8''), 15.9 (C-9'').

1,3,5-Trihydroxy-6',6'-dimethyl-2*H*-pyrano(2',3:6,7)xanthone (**26**)

^1^H NMR (600 MHz, DMSO-*d*_6_): *δ*_H_ 13.04 (1H, s, OH-1), 6.40 (1H, d, *J* = 1.7 Hz, H-4), 6.17 (1H, d, *J* = 1.7 Hz, H-2), 6.58 (1H, d, *J* = 9.9 Hz, H-1'), 5.89 (1H, d, *J* = 9.9 Hz, H-2'), 1.46 (6H, s, CH_3_-4', 5').

^13^C NMR (150 MHz, DMSO-*d*_6_): *δ*_C_ 179.5 (C-9), 165.3 (C-3), 162.8 (C-1), 157.2 (C-4a), 146 (C-6 and 10a), 133.1 (C-5), 131.7 (C-2'), 121.1 (C-1'), 118.3 (C-7), 113.8 (C-8a), 112.1 (C-8), 101.6 (C-9a), 98 (C-2), 94 (C-4), 77.7 (C-3'), 27.9 (C-4' and 5').

Pruniflorone N (**27**)

^1^H NMR (600 MHz, DMSO-*d*_6_): *δ*_H_ 12.91 (1H, s, OH-1), 10.57 (1H, s, OH-5), 7.55 (1H, d, *J* = 8.0, 1.4 Hz, H-8), 7.27 (1H, t, *J* = 8.0 Hz, H-7), 7.34 (1H, dd, *J* = 8.0, 1.4 Hz, H-6), 6.20 (1H, s, H-2), 5.42 (1H, d, *J* = 6.5 Hz, H-1′), 1.92 (1H, m, H-2′), 1.82 (1H, m, H-2′), 1.64 (3H, s, CH_3_-4′), 1.53 (3H, s, CH_3_-5′).

^13^C NMR (150 MHz, DMSO-*d*_6_): *δ*_C_ 180.8 (C-9), 160.3 (C-1), 160.3 (C-3), 155.3 (C-4a), 146.6 (C-5), 144.9 (C-10a), 124.3 (C-7), 120.6 (C-8a), 120.1 (C-6), 114.2 (C-8), 109.5 (C-4), 103.6 (C-9a), 98.8 (C-2), 92.9 (C-1'), 45.5 (C-2'), 31.8 (C-3'), 28.3 (C-5'), 28.2 (C-4').

Xanthone V_1_ (**28**)

^1^H NMR (600 MHz, DMSO-*d*_6_): *δ*_H_ 13.49 (1H, s, OH-1), 7.51 (d, *J* = 8.7 Hz, H-8), 6.94 (d, *J* = 8.7 Hz, H-7), 3.46 (2H, d, *J* = 7.3 Hz, H-1''), 5.24 (1H, t, *J* = 7.3 Hz, H-2''), 1.78 (3H, s, CH_3_-4''), 1.62 (3H, s, CH_3_-5''), 6.58 (1H, d, *J* = 9.9 Hz, H-1'), 5.73 (1H, d, *J* = 9.9 Hz, H-2'), 1.41 (6H, s, CH_3_-4', 5').

^13^C NMR (150 MHz, DMSO-*d*_6_): *δ*_C_ 180.3 (C-9), 157.1 (C-1), 155 (C-3), 153.6 (C-4a), 152.3 (C-6), 146.4 (C-10a), 132.7 (C-5), 130.9 (C-3''), 128 (C-2'), 122.2 (C-2''), 116 (C-8), 114.9 (C-1'), 113.1 (C-7), 112.9 (C-8a), 107.1 (C-4), 103.6 (C-2), 102 (C-9a), 77.9 (C-3'), 27.8 (C-4'), 27.8 (C-5'), 25.6 (C-5''), 21 (C-1''), 17.8 (C-4'').

Osajaxanthone (**29**)

^1^H NMR (600 MHz, DMSO-*d*_6_): *δ*_H_ 13.28 (1H, s, OH-1), 10.04 (1H, s, OH-7), 7.49 (1H, d, *J* = 9.0 Hz, H-5), 7.41 (1H, d, *J* = 3.0 Hz, H-8), 7.30 (1H, dd, *J* = 9.0, 3.0 Hz, H-6), 6.62 and 5.79 (each 1H, d, *J* = 10.0 Hz, H-1′, H-2′), 1.44 (6H, s, CH_3_-4′, 5′), 6.43 (1H, s, H-4).

Cochinchinone I (**30**)

^1^H NMR (600 MHz, DMSO-*d*_6_): *δ*_H_ 13.22 (1H, brs, 1-OH), 10.04 (1H, brs, 7-OH), 7.50 (1H, d, *J* = 9.0 Hz, H-5), 7.41 (1H, d, *J* = 3.0 Hz, H-8), 7.31 (1H, dd, *J* = 9.0, 3.0 Hz, H-6), 6.63 (1H, d, *J* = 10.0 Hz, H-1'), 5.78 (1H, d, *J* = 10.0 Hz, H-2'), 1.44 (6H, CH_3_-4', 5'), 3.40 (2H, d, *J* = 7.1 Hz, H-1''), 5.17 (1H, t, *J* = 7.1 Hz, H-2''), 1.92 (2H, m, H-4''), 1.97 (2H, d, *J* = 6.8 Hz, H-5''), 4.95 (1H, t, *J* = 6.8 Hz, H-6''), 1.44 (3H, s, CH_3_-8''), 1.83 (3H, s, CH_3_-9''), 1.47 (3H, s, CH_3_-10'').

^13^C NMR (150 MHz, DMSO-*d*_6_): *δ*_C_ 180.4 (C-9), 157.4 (C-3), 154.7 (C-1), 154.1 (C-4a), 153.8 (C-10a), 149 (C-7), 134.5 (C-3''), 130.6 (C-7''), 128.3 (C-2'), 124.2 (C-6''), 124 (C-6), 121.8 (C-2''), 120.1 (C-8a), 119.2 (C-5), 114.8 (C-1'), 107.8 (C-8), 106.7 (C-4), 103.5 (C-2), 102.5 (C-9a), 78.1 (C-3'), 40 (C-5''), 27.8 (C-4' and 5'), 26 (C-4''), 25.3 (C-8''), 20.9 (C-1''), 17.4 (C-10''), 16 (C-9'').

1,7-Dihydroxy-4-(3,7-dimethylocta-2,6-dienyl)-5'-(1-hydroxy-1-methylethyl)-4',5'-dihydrofuro[2',3':3,2]-xanthone (**31**)

^1^H NMR (600 MHz, DMSO-*d*_6_): *δ*_H_ 13.08 (1H, brs, 1-OH), 10.11 (1H, brs, 7-OH), 7.48 (1H, d, *J* = 9.1 Hz, H-5), 7.41 (1H, d, *J* = 2.8 Hz, H-8), 7.30 (1H, dd, *J* = 9.1, 2.8 Hz, H-6), 4.77 (1H, t, *J* = 8.3 Hz, H-2′), 4.72 (1H, s, OH-3′), 3.11 (2H, d, *J* = 8.3 Hz, H-1′), 1.17 (3H, s, CH_3_-5′), 1.17 (3H, s, CH_3_-4′), 3.40 (2H, d, *J* = 6.7 Hz, H-1''), 5.23 (1H, t, *J* = 6.7 Hz, H-2''), 1.99 (2H, m, H-4''), 1.93 (2H, m, H-5''), 4.97 (1H, t, *J* = 7.1 Hz, H-6''), 1.47 (3H, s, CH_3_-8''), 1.80 (3H, s, CH_3_-9''), 1.45 (3H, s, CH_3_-10'').

^13^C NMR (150 MHz, DMSO-*d*_6_): *δ*_C_ 180.1 (C-9), 165.6 (C-3), 154.8 (C-1), 154.3 (C-4a), 154 (C-7), 149.1 (C-10a), 134.8 (C-3''), 130.6 (C-7''), 124.5 (C-6), 123.9 (C-6''), 121.5 (C-2''), 120.1 (C-8a), 119 (C-5), 107.8 (C-8), 107.3 (C-2), 102.8 (C-9a), 101.2 (C-4), 91.2 (C-2'), 70.2 (C-3'), 39.5 (C-4''), 26.3 (C-1'), 26.1 (C-4'), 26 (C-5''), 25.3 (C-8''), 24.3 (C-5'), 21.6 (C-1''), 17.5 (C-10''), 15.9 (C-9'').
